# Steroid-induced Glaucoma: An Avoidable Irreversible Blindness

**DOI:** 10.5005/jp-journals-l0028-1226

**Published:** 2017-08-05

**Authors:** Sonia Phulke, Sushmita Kaushik, Savleen Kaur, SS Pandav

**Affiliations:** 1 Senior Resident, Department of Ophthalmology, Postgraduate Institute of Medical Education & Research, Chandigarh, India; 2 Professor, Advanced Eye Centre, Postgraduate Institute of Medical Education & Research, Chandigarh, India; 3 Senior Research Associate, Advanced Eye Centre, Postgraduate Institute of Medical Education & Research, Chandigarh, India; 4 Senior Research Associate, Advanced Eye Centre, Postgraduate Institute of Medical Education & Research, Chandigarh, India

**Keywords:** Glaucoma, Intraocular pressure, Ocular hypertension, Steroids.

## Abstract

Steroids are a group of anti-inflammatory drugs, commonly used to treat ocular and systemic conditions. Unmonitored use of steroids especially in eye drop formulations is common in situations when it is easily available over-the-counter, resulting in undesirable side effects.

Among the ocular side effects, cataract and glaucoma are common. Steroid-induced ocular hypertension was reported in 1950, when long-term use of systemic steroid was shown to increase the intraocular pressure (IOP). Chronic administration of steroids in any form with raised IOP can cause optic neuropathy resulting in steroid-induced glaucoma.

This review describes the pathophysiology and epidemiology of steroid-induced glaucoma, recognition of side effects, and principles of management. The purpose is to familiarize all clinicians with the potential dangers of administering steroids without monitoring the eye and the dangers of irreversible blind -ness in some instances of habitual self-prescription by patients.

**How to cite this article:**

Phulke S, Kaushik S, Kaur S, Pandav SS. Steroid-induced Glaucoma: An Avoidable Irreversible Blindness. J Curr Glaucoma Pract 2017;11(2):67-72.

## INTRODUCTION

Corticosteroids are a class of anti-inflammatory drugs commonly used to treat various ocular and systemic conditions. The use of steroids can lead to significant ocular side effects. Intraocular pressure (IOP) elevation following steroid use is well- documented.^1^ Steroids are known to induce ocular hypertension when administered with topical, periocular, and even systemic or inhalational routes. There are many instances where one can avoid the use of steroids and switch over to nonsteroidal/anti-inflammatory alternatives and where it is not possible, monitoring the IOP is essential irrespective of the dose and duration of the steroid use. The ocular hypertensive response is fairly reversible and if intervened at the right time can prevent vision threatening complication which is especially important in children.

## ETIOLOGY

Steroid-induced glaucoma is a form of open angle glaucoma. The precise mechanism for IOP elevation after steroid intake is not very clear, but primarily it occurs due to reduced facility of aqueous outflow. The following are proposed theories for steroid-induced raised IOP:

- Steroid causes stabilization of lysosomal membranes and accumulation of polymerized glycosamino-glycans (GAGs) in the trabecular meshwork. These polymerized GAGs become hydrated, producing "biologic edema" and increased outflow resistance.^2-4^
  Glucocorticoids also increases the expression of extracellular matrix protein fibronectin, GAGs, elastin, and laminin within the trabecular meshwork cells which leads to increased trabecular meshwork resistance.^5,6^ Ultrastructurally, in steroid-induced glaucoma, there is accumulation of basement membrane like material staining for type IV collagen.^7,8^
  Corticosteroids cause inhibition of phagocytotic properties of endothelial cells lining the trabecular meshwork which leads to accumulation of aqueous debris.^9^
- Glucocorticoids have been shown to alter the trabecu -lar meshwork cell morphology by causing an increase in nuclear size and DNA content.^10^ Zhang et al^11^ have suggested that FKBP06-binding immunophilin FKBP51 mediates nuclear transport of the human glucocorticoid receptor beta, suggesting that this may play a role in increased glucocorticoid responsiveness.
- Recent case series has shown that trabecular outflow obstruction can occur due to crystalline steroid particles after receiving intravitreal triamcinolone ace-tonide (IVTA) injections for diabetic macular edema.^12^
- Glucocorticoid decreases the synthesis of prostaglandin, which regulates the aqueous outflow.^13^
- *Genetic influence:* Several genes have been found to be associated with both protective and damaging glucocorticoid-treated trabecular meshwork cells. Glucocorticoids may exert their effect by increased expression of MYOC [Trabecular meshwork-induced glucocorticoid response (TIGR)] gene at locus GLC1A.^13^ However, recent studies in monkeys failed to show a significant link between myocilin gene and steroid-induced ocular hypertension.^14^ Other than myocilin gene alpha1 antichymotrypsin, pigment epithelium-derived factor, cornea-derived transcript 6, prostaglandin D_2_ synthetase, growth arrest specific 1, decorin, insulin-like growth factor binding protein 2, ferritin light chain, and fibulin-1C are other genes which have been postulated to play a role in steroid responsiveness but more studies are required to confirm their role in steroid responsiveness.^15-17^

**Table 1:** **Table 1:** Studies of Becker and Armaly demonstrating IOP response to steroid^22,23-25^

*Feature/Parameter*				*Becker*		*Armaly*	
Frequency				QID^a^		TDS^b^	
Duration				6 weeks		4 weeks	
Parameter				Final IOP		IOP change	
Type of responder		Low		<20 mm Hg (58%)		<6 mm Hg (66%)	
		Intermediate		20-31 mm Hg (36%)		6-15 mm Hg (29%)	

a: QID: quater indie; b. TDS: ter die sumendum

**Table 2:** **Table 2:** Steroid responsiveness in nonglaucomatous, glaucomatous, and glaucoma suspect eyes

		*Incidence of steroid responsiveness (%)*					
		*Responders*					
*Population*		*High*		*Intermediate*		*Nonresponders*	
General population^22^		5		*35*		60	
POAG^26^		90		10		0	
Siblings of POAG^27^		30		50		20	
Offspring of POAG^28^		25		70		5	

## EPIDEMIOLOGY

Steroid-induced IOP elevation can occur in any age group. Most studies describing steroid-induced glaucoma have focused on adults. Children are also known to have a severe ocular hypertensive response to topical steroids when compared to adults and significant IOP elevation has also been reported in infants treated with nasal and inhalational steroids.^18,19^

In a previously reported study, the authors found steroid-induced glaucoma to account for one-fourth of all acquired glaucomas in children.^20^ Increasing trends of steroid-induced glaucoma in children over the last few years have been reported, probably signifying increasing use of unmonitored steroid use.^21^

There are many ill understood facts regarding pressure response to steroids in different individuals, precise distribution of steroid responders in the general population and the reproducibility of these responses, and the hereditary influences. In the classic studies of Becker^22^ and Armaly,^23-25^ they categorized the general population into high, intermediate and low response according to their pressure response to topical steroid drops (Table 1).

## RISK FACTORS

There are certain conditions which have been considered to increase the risk of developing steroid-induced glaucoma.

Patient-related factors:

- Patients with primary open angle glaucoma (POAG)
  – Approximately 30% of glaucoma suspects and 90% of POAG might have an ocular hypertensive response to a 4-week course of topical dexametha-sone 0.1% .^26^ Normal individuals classified as high steroid responders are more likely to develop POAG. First degree relatives of POAG patients are also considered to have a higher risk of being a steroid responder. Table 2 highlights the association between POAG and steroid responsiveness
- High myopia^29^ or eyes with history of penetrating keratoplasty or refractive surgeries like photorefractive keratectomy,^30^ laser in situ keratomileusis (LASIK),^31^ and Descement’s Stripping Endothelial Keratoplasty (DSEK).^32^ Steroid-induced glaucoma after refractive surgery is masked by falsely low IOP measurement due to thin central corneal thickness, ocular rigidity changes, corneal edema, and fluid accumulation beneath the LASIK flap^33,34^
- Very young (<10 years of age) and older adults (bimodal distribution)
- Diabetes mellitus^35^ or connective tissue disease (especially rheumatoid arthritis)^36^
- Eyes with pigment dispersion syndrome^37^ or traumatic angle recession^38^
- Endogenous hypercortisolism.^39^

## ROUTES OF ADMINISTRATION

- Exogenous corticosteroids are more likely to cause IOP elevation. However, endogenous corticosteroids can also cause this condition, as enumerated in Table 3.
- *Topical therapy:* IOP rise after steroid therapy occurs more frequently with topical administration than systemic administration. The IOP rise may occur with drops or ointment applied directly to the eye or over the skin of the eyelids.^40,41^
- *Periocular therapy:* Periocular injections of repository corticosteroid is the most dangerous form of steroid because of their prolonged duration of action.^42^ Intraocular pressure elevation may occur in response to subconjunctival, subtenon, or retrobulbar injection of steroids.^43^ A patient’s response to earlier topical steroid therapy does not predict their response to periocular steroid.^44^
- *Intravitreal therapy:* Intraocular pressure elevation with intravitreal depot steroid has been reported in a large percentage of patients. Ozurdex is a slow release intravitreal implant of dexamethasone used in the treatment of macular edema secondary to vein occlusions and for the treatment of uveitis. Previous studies have indicated that >10 mm Hg IOP increase from baseline occurs in 12.6% of patients after the first treatment and 15.4% after the second dose of Ozurdex. As dexamethasone-induced ocular hypertension is transient and it is a more water-soluble steroid than triamcinolone or fluocinolone acetonide, IOP may be better controlled without causing any ocular complica-tions.^45^ Intravitreal injection of triamcinolone on the contrary can increase IOP by several mm Hg in about 50% of patients, within 2 to 4 weeks after the start of treatment and even may rise more rapidly in some eyes (e.g., in eyes that have pseudophakia or have undergone vitrectomy).^46,47^ Smithen reported that among the nonglaucoma patients treated with intravitreal triam-cinolone, 60% of patients with baseline IOP >15 mm Hg showed a rise in IOP following injection compared to 22% of those who had baseline IOP <15 mm Hg.^48^
- Systemic therapy of steroids is the least likely route to causing IOP elevation.^49^ However, if the pressure rises, this response does not correlate with the dosage or duration of treatment.^50^
- *Another routes:* There exists a strong association between inhaled corticosteroid and the presence of ocular hypertension and the risk increased with higher dose and more puffs in patients with family history of glaucoma.^51^

**Table 3:** **Table 3:** Routes of administration leading to steroid-induced glaucoma

*Exogenous*		*Topical*		*Systemic^49,50^*		*Periocular*	
		Eyedrops^26^		Oral		Subtenon injections,	
		Ointments^40^		Topical skin		Subconjunctival or retrobulbar	
				Preparations		injections	
		Intravitreal injection^48^		Inhalational^51^			
		Intravitreal implants^45^		Injection			
Endogenous^39^		Adrenal hyperplasia, adenoma, carcinoma					
		Ectopic ACTH syndrome					

ACTH: Adrenocorticotropic hormone

In general, the pressure-inducing effect of steroids is directly proportional to its anti-inflammatory potency.^52^ However, the pressure-inducing potency is related to the dosage of the drug used (0.01% of betamethasone in high topical steroid responders caused significantly less pressure elevation than with 0.1%).^53^

## DURATION OF STEROID ADMINISTRATION

In steroid responsive patients, IOP elevation usually develops within the first few weeks of steroid administration. However, it can be elevated within an hour or many years after chronic steroid use. After steroid is discontinued, IOP usually normalizes within 1 to 4 weeks.

## CLINICAL FEATURES

Patients usually present with very few symptoms like that of POAG. Signs and symptoms vary with the age of the patients. Infants may present with features of primary congenital glaucoma like watering, blepharospasm and photophobia. Teenagers present like developmental or juvenile open angle glaucoma. Adult patients present with raised IOP, normal and open angles on gonioscopy, optic disk cupping, and visual field defects. Other ocular findings are increased corneal thickness, corneal ulcers, and posterior subcapsular cataract. Steroid-induced glaucoma in vernal keratoconjunctivitis (VKC) is a common complication as patients require long-term treatment and steroids are often used to provide relief of symptoms.^54^ Long-term study by Bonini et al have reported 2% incidence of steroid-induced glaucoma in VKC.^55^

## DIFFERENTIAL DIAGNOSIS

- Primary open angle glaucoma
- Normal tension glaucoma
- Juvenile open angle glaucoma
- Uveitic glaucoma
- Glaucomatocyclitic crises

## MANAGEMENT

- Discontinuation of the use of the steroid is the first line of management. In the majority of cases, steroid-induced acute rise of IOP typically normalizes within days of stopping the steroid and chronic forms take 1 to 4 weeks. In rare cases, IOP remains elevated, for which antiglaucoma medications or surgery may become necessary. The duration of steroid therapy also appears to influence the reversibility of the IOP elevation.^56^
- Sometimes steroids cannot be withheld in view of sys -temic need of patients, in such cases corticosteroids like betamethasone, prednisolone, and dexametha-sone can be substituted with nonadrenal steroids like rimexolone, loteprednol etabonate, fluorometholone, and medrysone. These drugs have useful anti-inflammatory properties with significantly less IOP elevation effect. Fluorometholone 0.1% is more effective in treating anterior segment ocular inflammation; however, significant IOP elevation has also been observed with this drug.^57^ Fluorometholone 0.25% is also less likely to increase IOP in steroid respond-ers, but increased concentration does not appear to enhance the drug’s anti-inflammatory effect.^58^
- Use of repository steroid is particularly dangerous due to their prolonged duration of action. It is better to inject this depot in the inferior quadrant and in anterior location to preserve the superior site for future glaucoma filtering surgeries, if needed in future.
- Depot steroid-induced IOP elevation can be effectively controlled after excision.^44,46^ If it is not possible to excise them, filtering surgery is advised to control IOP. Glaucoma due to intravitreal triamcinolone injections is medically managed, although role of pars plana vitrectomy has also been documented.^59^
- Another treatment modality could be substituting with steroid-sparing agents or topical non-steroidal anti-inflammatory drugs (NSAIDs) like flurbipro-fen, bromfenac, ketorolac, and nepafenac. These NSAIDs primarily act as cyclooxygenase inhibitors, may be effective in anterior segment ocular inflammation.^60-63^
- *Glaucoma therapy:* When medical management fails to control the IOP, selective lasers or argon laser trabeculoplasty can be tried in, particularly in eyes treated with intravitreal triamcinolone.^64,65^ Anterior subtenon injection of anecortave acetate, which is a synthetic derivative of cortisol used in the treatment of age-related macular degeneration,^66^ has shown to be effective in reducing IOP in these patients.^67^ Surgery like trabeculotomy,^68^ trabeculectomy with or without antimetabolites or glaucoma drainage device are reserved for medically uncontrolled or intractable glaucoma.

## SOCIAL IMPACT OF THE DISEASE

Steroid-induced glaucoma is an iatrogenic disease, which can be prevented. Increased use of steroids for almost any ocular as well as extraocular pathology has led to their overuse. An increasing amount of cases with steroid-induced glaucoma reported in the literature have made this a global issue. There is an enormous disability caused by steroid-induced glaucoma in patients of all age groups. Cheap and easily available dexamethasone eye drop over the counter forms the primary prescription of many ophthalmologists and chemists. Use of such easily available steroids reduces the symptoms of the primary pathology, leading to a sense of betterment, which usually results in patients continuing to use steroids unmonitored over long periods of time. Steroid-induced glaucoma is a significant health problem in the pediatric age group, but it responds well to the withdrawal of steroids and medical treatment. The most effective way of treating this ocular hypertensive response appears to be the cessation of the steroid therapy. Self-medication should be avoided and discouraged under all circumstances. Wherever possible, other alternatives like immunosuppressant should be used where the risk of steroid-induced ocular hypertension or glaucoma is suspected.

## CONCLUSION

Steroid-induced glaucoma is an iatrogenic and preventable disease. The unwarranted and irrational use of steroids especially in developing countries by local medical practitioners as well as unmonitored self-use by patients themselves points to a lack of awareness about the disease. Prevention of steroid-induced glaucoma can be achieved with a few simple precautions. Identification of risk factors (like POAG, family history, high myopia, diabetes mel-litus, and connective tissue disorder) and monitoring for ocular hypertension can decrease the development of irreversible glaucomatous optic neuropathy. For discouraging self-medication, monitoring for IOP after prescription of steroids in any form and prompt management is essential. These practical and safe guidelines for the use of steroids should be followed by all doctors.
